# Evaluation of rheological and optical properties plus stability of beverage cloud emulsions prepared with corn oil, gum rosin, and modified starch

**DOI:** 10.1002/fsn3.3115

**Published:** 2022-11-17

**Authors:** Soghra Nasrolahi, Jalal Sadeghizadeh‐Yazdi, Mohammad Hassan Ehrampoush, Farzan Madadizadeh, Elham Khalili

**Affiliations:** ^1^ Master of Food Science and Technology, School of Public Health Shahid Sadoughi University of Medical Sciences Yazd Iran; ^2^ Department of Food Science and Technology, School of Public Health Shahid Sadoughi University of Medical Sciences Yazd Iran; ^3^ Environmental Science and Technology Research Center, School of Public Health Shahid Sadoughi University of Medical Sciences Yazd Iran; ^4^ Department of Environmental Health Engineering, School of Public Health Shahid Sadoughi University of Medical Sciences Yazd Iran; ^5^ Department Biostatistics, School of Public Health Shahid Sadoughi University of Medical Sciences Yazd Iran; ^6^ Department of Food Hygiene and Safety, School of Public Health Shahid Sadoughi University of Medical Sciences Yazd Iran

**Keywords:** corn oil, gum rosin, modified starch, rheological properties, stability

## Abstract

Rheological and optical properties as well as stability of beverage cloud emulsion prepared with corn oil, gum rosin (EG), and modified starch were evaluated in model juices. The emulsions were prepared with three levels of modified starch (6%, 12%, and 18% w/w), corn oil (5%, 7%, and 9% w/w), and gum rosin (1%, 3%, and 5% w/w). Experiments were designed using the Box–Behnken design. The analysis of variance (ANOVA) was used to evaluate the significance of the experimental factors and the factors were then optimized using response surface methodology (RSM). The stability of emulsions was measured through ring formation in both the primary emulsion and the model beverage as a function of storage time. Also, the effect of heat treatment was examined on the stability of emulsions in model beverages. The results revealed that heat treatment did not cause the formation of an observable ring in the model juice containing stabilized starch emulsion. Rheological examinations of the stable emulsion samples showed a pseudoplastic and time‐independent non‐Newtonian behavior. The optimum emulsion sample consistency coefficient was 0.46 Pa.s^n^ and the flow behavior index was 0.88. The apparent viscosity of the optimum emulsion sample based on Herschel–Bulkley model at shear rate of 100 s^−1^ was 0.0439 Pa.s. The results indicated that the concentration of modified starch, gum rosin, and corn oil has a significant effect on the stability and creaminess of the emulsion. In general, with an increase in the percentage of modified starch, the stability rises while the rate of creaminess decreases (*p* < 0.05). Furthermore, elevation of the concentration of corn oil had a significant effect on the opacity of emulsions and the final product (*p* < 0.05).

## Graphical abstract

Starch Modified due to the absorption of water in the continuous phase, as an emulsifier plays an effective role in creating suitable texture and stability of the emulsion. The superior functionality of Starch Modified permits the preparation of very fine stable emulsions, often at reduced pressures or shear which results in additional energy savings. Compared to Arabic gum, smaller amounts of Starch Modified are needed for stabilization of beverage emulsion. Gum Rosin has been used as a weighting agent in the preparation of beverage emulsions. Both modified starch and corn oil have a significant effect on the cloudy appearance of lemon drink.

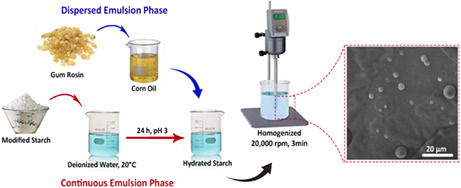

## 1 INTRODUCTION

The sensory and physicochemical properties of emulsion‐based food products are strongly influenced by the properties of their components, such as concentration, particles size, electric charge, and the reactions between them. The diameter of the drops of emulsion food products varies within 0.1–100 μm. Such systems have minimal stability due to the surface tensile force between the components of these two liquids. Emulsions consist of two phases, including dispersed phase and continuous phase. The boundary between the two phases is called the middle boundary. The emulsification process involves dispersion of two impermeable liquids in the presence of emulsifier where amphiphilic molecules are absorbed at the interface of water and oils and reduce the surface tension (Buffo et al., 2002). Emulsions appear cloudy and turbid because when light passes through the emulsion, a large amount of it is scattered by the intermediate phase. The color and appearance characteristics of the emulsion depend on the size and number of dispersed phase droplets as well as the difference in the refractive index of the two phases. For example, when the droplet size is larger than 1 μm, the environment looks milky, but when the size is less than 0.05 μm, the environment becomes transparent. Obviously, the difference between the refractive index of the two phases leads to reduced transparency. Emulsions are thermodynamically unstable and separated over time. In this regard, it is necessary to use emulsifiers to stabilize the oil–water interface and maintain the dispersed oil droplets in the aqueous phase. Emulsifiers are surfactant bipolar molecules that contain soluble and insoluble parts in water. Hence, they can be absorbed at the oil–water interface during emulsification and prevent reaccumulation of droplets. Emulsifiers also reduce the surface tension in oil–water interface. Thus, they facilitate oil emulsification. Dispersion of the oil phase in smaller oil droplets requires mechanical forces. During homogenization, decomposition (breaking) of the droplets occurs, and as a result, the droplet size decreases, which leads to stabilization of the emulsions. This breaking is usually done by high‐energy methods such as high‐speed mixers, high‐pressure valve homogenizers, or microfluidizers (McClements, 2004; McClements & Rao, 2011).

Hydrocolloids are the stabilizers mainly used for the stability of emulsion beverage. Hydrocolloids with a high solubility in cold water, low viscosity, high emulsification capacity, without concentrating effect, are suitable for this purpose. Bipolar polysaccharides such as Arabic gum and modified starch are the most common type of such hydrocolloids (Moreira et al., 2012). These hydrocolloids are surfactants and are absorbed at the interface between oil and water, and facilitate the production of small droplets by reducing surface tension during homogenization. The formation of an oily ring is a major quality defect in citrus beverages, as well as other beverages. Formation of a relatively white ring around the neck of the dish or glossy oil slime on top of the product is a result of gravitational separation (Bohlin, 1980). For preventing such instability mechanisms, the proper selection of emulsifier and homogenization mechanism is very vital. One of the most important challenges in the formulation of beverage emulsions is the assurance of their physicochemical stability (Peressini et al., 1998). The most suitable alternative for Arabic gum in emulsion beverages is modified starch. These compounds are a group of starch derivatives where the lipophilic groups substituted on the main chain confer the molecule an amphiphilic characteristic. Starch derivatives can be converted to low‐viscosity starch via acid hydrolysis or by enzymatic digestion. Starches modified in aqueous solutions are relatively anionic and have a surface activity similar to Arabic gum. One of the advantages of the modified starch is that it has a higher degree of purity than Arabic gum and the impurities with it are very small. It also dissolves in cold water and has no distinct taste. Furthermore, it has good stability against changes in temperature and pH. Compared to Arabic gum, smaller amounts are needed for stabilization. In this regard, the aim of this study is to investigate rheological properties and stability of beverage cloud emulsions prepared with corn oil, gum rosin, and modified starch.

## 2 MATERIALS AND METHODS

### 2.1 Materials

Corn oil was prepared under the brand name ZAR Oil from the grocery store, modified starch (Purity Gum Be) from the National Starch & Chemical GmbH (Canada), gum rosin from India (Sood Paper & Allied Chemicals), citric acid from Jovin Iran Co., sodium benzoate from Dalian Future International Co. (China), lemon concentrate from Dohler Co. (Germany), fructose syrup from Zar Fructose Co. (Iran), sugar from Karun Iran Agriculture & Industry Co., and deionized water from Zolal Shimi Co. (Iran).

### 2.2 Beverage emulsion preparation

The response surface methodology (RSM) was used to evaluate the stability changes based on operational parameters including modified starch concentration, corn oil concentration, and gum rosin. Different combinations of three variables were designed using Box–Behnken design, where 17 runs with three factors at three levels were generated using Design‐Expert version 13.0.5.0 × 64. For preparing a continuous emulsion phase, modified starch at different levels (6%, 12%, and 18% w/w) was dissolved in deionized water at 20°C which contained sodium benzoate (3 ppm). Then, the mixtures were kept at room temperature for 24 h to hydrate the starch. Next, the pH of the continuous phase was set at 3 using citric acid (Taherian et al., 2007). Also, for preparing the dispersed emulsion phase, gum rosin at three levels (1%, 3%, and 5% w/w) was added to corn oil (5%, 7%, and 9% w/w) and kept overnight (Kanyuck, 2017). Then, the dispersed phase was added to the continuous phase. In this step, a mixer with a maximum speed of 19 *g* for 10 min was employed to produce pre‐emulsion. The oil droplet size was reduced using a laboratory homogenizer (Silent crusher M‐Germany) at a maximum speed of 2907 *g* for 3 min. A mechanical stirrer with a maximum speed of 19 *g* for 10 min was used to mix the dispersed and continuous phases (prehomogenization). The oil droplet size was reduced using a laboratory homogenizer (Silent crusher M‐Germany) at a maximum speed of 2907 *g* for 3 min. Once the homogenization finished, the samples were packed in suitable containers and kept at room temperature for further experiments.

### 2.3 Model juice preparation

The model juice was prepared by mixing the following compounds at the mentioned concentrations: purified water (89.7%), fructose syrup (5.6%), sugar (4%), citric acid (0.25%), sodium benzoate (0.015%), and lemon concentrate (1%). The mixture was stirred by a home mixer at a speed of 182 *g* for 10 min. A 0.05% emulsion was mixed with the juice model. The pH of the lemon juice prepared with the emulsion remained unchanged. Lemon beverage was pasteurized under two different conditions, including 70°C for 30 min and 90°C for 3 min, in a hot water bath at atmospheric pressure without stirring (Chaudhari et al., 2015).

### 2.4 Rheological characteristics

The Physica MCR 301 rotary rheometer (Austria, Anton Paar) equipped with a Peltier Plate temperature controller and a water circulator with a sensitivity of 0.01 using concentric cylinder geometry CC27 was utilized to evaluate the rheological characteristics (40 mm diameter and 1 mm gap). Also, to prevent solvent evaporation, the samples were covered with solvent traps during the experiment. Rheological data were analyzed using software Rheoplus, version V3.4. Flow curves were obtained at shear rates of 0.01–1000 s^−1^ within 8 min. Experimental flow curves were fitted to the Herschel–Bulkley model.

$$y=y_{0}+k{\text{34𝛾}}^{n}$$

where *y*_0_ is the yield stress, *k* denotes the consistency index, and *n* shows the flow behavior index. Consistency coefficient (*k*) and flow behavior index (*n*) values plus apparent viscosity values (at the shear rate of 100 s^−1^) were determined (Ramaswamy et al., 2020). Dynamic measurements were conducted to describe the rheological properties of emulsions more distinctly. The strain sweep test was performed within the strain range of 0.01%–0.001%, at fixed frequency of 1 Hz, and 2°C. The results of strain sweep test were used to determine the limiting values of strain (γL), structural strength (G′ LVE), loss‐tangent value tan in the linear viscoelastic range (Tan δ LVE), yield stress at the limit of the LVE range (τy), and flow‐point stress (τf). The frequency sweep test was performed to determine the viscoelastic properties of the samples. For this purpose, a frequency sweep from 0.1 up to 10 Hz at 2°C was used at fixed oscillation stress (0.005%) where G′ (elastic modulus) and G″ (viscous modulus) were measured. Measurements were done in triplicate for each emulsion. The storage modulus (G′), loss modulus (G″), and phase angle (δ) are among the parameters that characterize a system in a dynamic rheological study. The linear viscoelastic region (LVR) was determined prior to each frequency sweep by performing stress sweeps to verify the linear relationship between stress and strain. G′ is a measure of the energy stored in a cycle of oscillation, while G″ is the measure of energy lost as viscous flow in a cycle of oscillation (Hesarinejad et al., 2014; Taherian et al., 2006).

### 2.5 Creaming stability measurement

The emulsion stability was reported as the ratio of emulsion phase volume to the total liquid volume (as percent) (Krstonosic et al., 2009). To determine the stability of the emulsion, immediately after preparation, 15 ml of it was poured inside a capped test tube and kept at 25°C for 7 days. During the storage time, some samples became diphasic. Next, the total height of the emulsion (HE) and the height of the serum layer (HC) were measured. The creaming index was calculated from the ratio of creaming volume to the emulsion samples total volume, without shaking. The results were expressed as a percentage of the total height of emulsions in the tube (HE) (Hosseini et al., 2015; Maravic et al., 2019).

$$\text{Creaming index}=100\times \left(\mathrm{HC}/\mathrm{HE}\right).$$

### 2.6 Emulsion opacity

The opacity of the samples was evaluated by diluting the emulsions (1:1000) and measuring the absorbance at 660 nm, using 4802 model spectrophotometer. The assessment of opacity was performed in triplicate (Taherian et al., 2006).

### 2.7 Statistical analysis

In this study, natural logarithm was used to stabilize the variance due to the scattering in the value of responses. According to Design‐Expert software version7, the quadratic model was the best option for fitting the experimental data and checking the accuracy of the model based on the coefficient of determination or power of the model, and lack of fit test was used. Also, multiple linear regressions with one variable were used to determine the effect of different variables on emulsion opacity. One‐way ANOVA was employed to compare the extent of adsorption in different samples. The level of significance in the analysis of tests was 5%. All statistical analyses were performed in three replications in SPSS software version 21.

## 3 RESULTS

The effect of modified starch, corn oil, and gum rosin concentration on emulsion stability was optimized using the RSM and based on the Box–Behnken design. Quadratic models were proposed by Design‐Expert software and used to fit the experimental data (Table 1). The following regression equations were fitted on it.

Stability=65.01000+8.44254×Modified Starch+15.47171×Corn Oil−0.26103×GumRosin+0.083333×Modified Starch×Corn Oil+0.018054×Modified Starch×GumRosin+9.72083E−003×Corn Oil×GumRosin−0.39169×Modified Starch^2–0.62322×Corn Oil^2–3.59850E−004×EsterGum.

**TABLE 1:** The quadratic model for optimal emulsion stability

Source	Sum of squares	*df*	Mean square	*F*	*p* > *F*
Model	11,579.59	9	1286.62	3.82	0.0154
A – modified starch	3842.58	1	3842.58	11.42	0.0118
B – corn oil	3444.92	1	3444.92	10.24	0.0151
C – gum rosin	410.98	1	410.98	1.22	0.3056
AB	36.00	1	36.00	0.11	0.0132
AC	469.37	1	469.37	1.39	0.0327
BC	136.07	1	136.07	0.40	0.0451
*A* ^2^	837.21	1	837.21	2.49	0.0158
*B* ^2^	2119.47	1	2119.47	6.30	0.0404
*C* ^2^	54.52	1	54.52	0.16	0.6993
Residual	2355.52	7	336.50		
Lack of fit	2204.12	3	734.71	19.41	0.7627
Pure error	151.40	4	37.85		
Cor total	13,935.12	16			

Based on the observed fit summary, the emulsion stability presented an *R* ^2^ value equal to 0.9108. It implied that 91.08% of the variance of the sample is attributed to the factors and only 8.92% occurred due to chance for the *R* ^2^ values. This indicated a good fit for emulsion stability (Table 2). Strain sweep test parameters were obtained for the optimal emulsion sample at 2°C and 1 Hz, as listed in Table 3. Table 4 reports the flow properties of emulsions at 2°C. The coefficient of determination, *R* ^2^, was 0.99 for all measurements. ANOVA results for the physicochemical properties of beverage emulsion are shown in Table 5. Table 6 deals with the effect of the concentration of modified starch, corn oil, and gum ester on the opacity of the emulsion.

**TABLE 2:** Fit summary and model check

Parameters	Stability of optimal emulsion
*R* ^2^	0.9108
Adjusted *R* ^2^	0.8136
Predicted *R* ^2^	0.6477
Adequate precision	6.065
Standard deviation	8.34
Mean	68.98
CV %	16.59

**TABLE 3:** Strain sweep test parameters obtained for the optimal emulsion sample at 2°C

Parameter	Mean ± SD	Parameter	Mean ± SD
G′ LVE (Pa)	3.41 ± 0.928	γL (%)	0.95 ± 0.083
G″ LVE (Pa)	3.30 ± 0.873	τy (Pa)	0.15 ± 0.014
Tan δ LVE	0.63	τf (Pa)	1.68 ± 0.029

**TABLE 4:** Comparison of flow properties of beverage emulsions at 2°C

Emulsion number				
Emulsion properties	3	4	7	10
*n*	0.93	0.88	0.88	0.88
*k*	0.06	0.46	0.46	0.46
y_0_	0.04	0.15	0.15	0.15
*η*	0.02	0.27	0.04	0.29
*R* ^2^	0.99	0.99	0.99	0.99

**TABLE 5:** ANOVA results for the physicochemical properties of beverage emulsion

Variable	Level (%)	Opacity (660 nm)	*p*	Creaming (%)	*p*	Stability	*p*	Ringing in bottle^*^	*p*
		Mean ± SD		Mean ± SD		Mean ± SD			
Modified starch	6	0.054 ± 0.001	0.02	74.36 ± 13.06	0.001	10.89 ± 26.76	0.001	++++	0.001
	12	0.057 ± 0.007		17.36 ± 5.26		5.26 ± 82.64		+	
	18	0.091 ± 0.002		19.32 ± 33.53		33.35 ± 80.67		−	
Corn oil	5	0.054 ± 0.007	0.06	26.86 ± 24.89	0.81	24.89 ± 73.13	0.83	++	0.28
	7	0.061 ± 0.001		33.89 ± 32.10		31.20 ± 26.76		+	
	9	0.089 ± 0.003		28.19 ± 30.55		30.55 ± 71.80		+	
Gum rosin	1	0.059 ± 0.001	0.61	29.64 ± 45.82	0.19	54.18 ± 29.64	0.19	+	0.34
	3	0.066 ± 0.002		23.79 ± 22.88		66.55 ± 31.20		+	
	5	0.066 ± 0.001		37.91 ± 35.42		65.70 ± 35.85		++	

* The number of + signs indicates the intensity and amount of oil ring formation.

**TABLE 6:** Determining the effect of the concentration of modified starch, corn oil, and gum ester on the opacity of the emulsion

Variable	Univariate model			Multiple		
	Unstandardized coefficients	Standardized coefficients	*p*	Unstandardized coefficients	Standardized coefficients	*p*
Modified starch	0.062	0.003	<0.001	0.062	0.003	<0.001
corn oil	0.492	0.008	0.003	0.492	0.008	<0.001
Gum rosin	0.122	0.002	0.491	–		–

## 4 DISCUSSION

### 4.1 Model validation and adequacy testing using normal plot of residual

**FIGURE 1:**
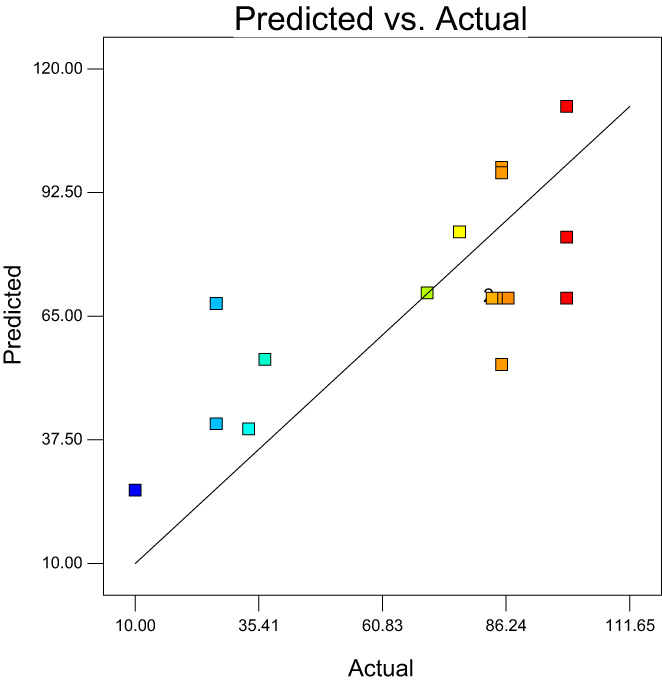
Predicted versus actual on emulsion stability

**FIGURE 2:**
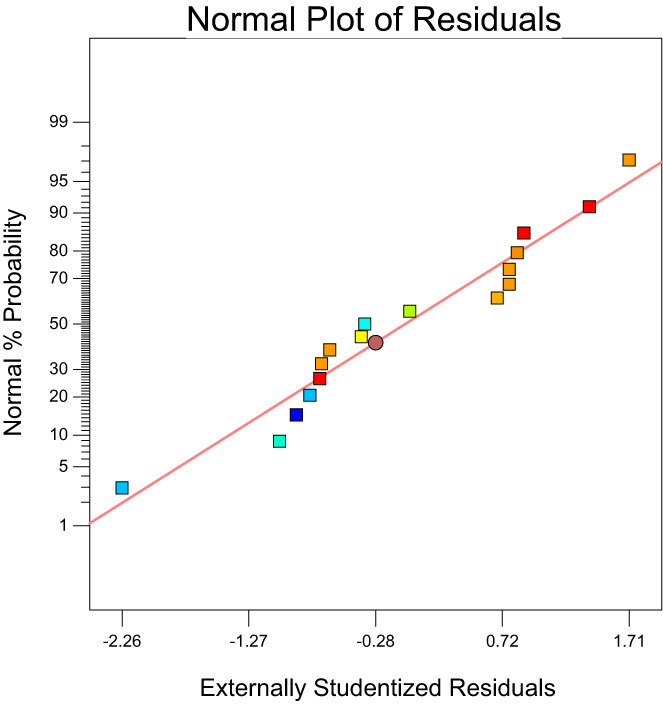
Normal plot of residual versus external studentized residuals on emulsion stability

**FIGURE 3:**
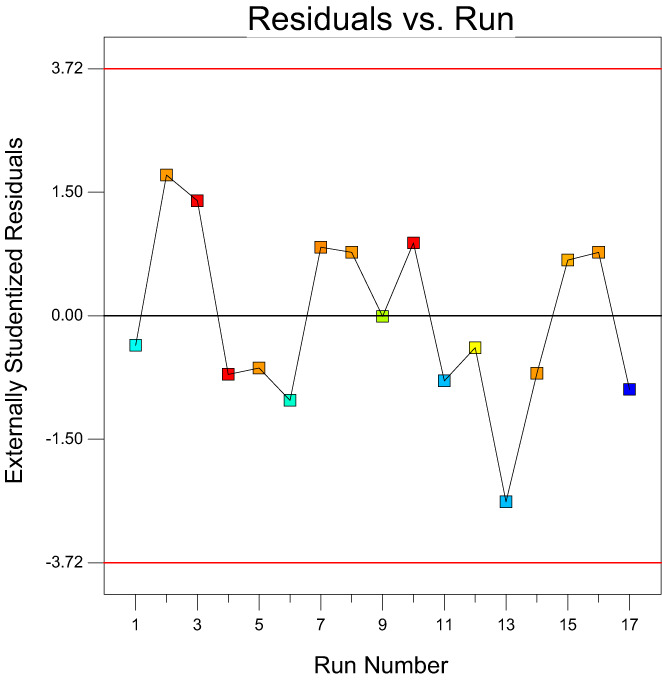
External studentized residual versus run number of emulsions

**FIGURE 4:**
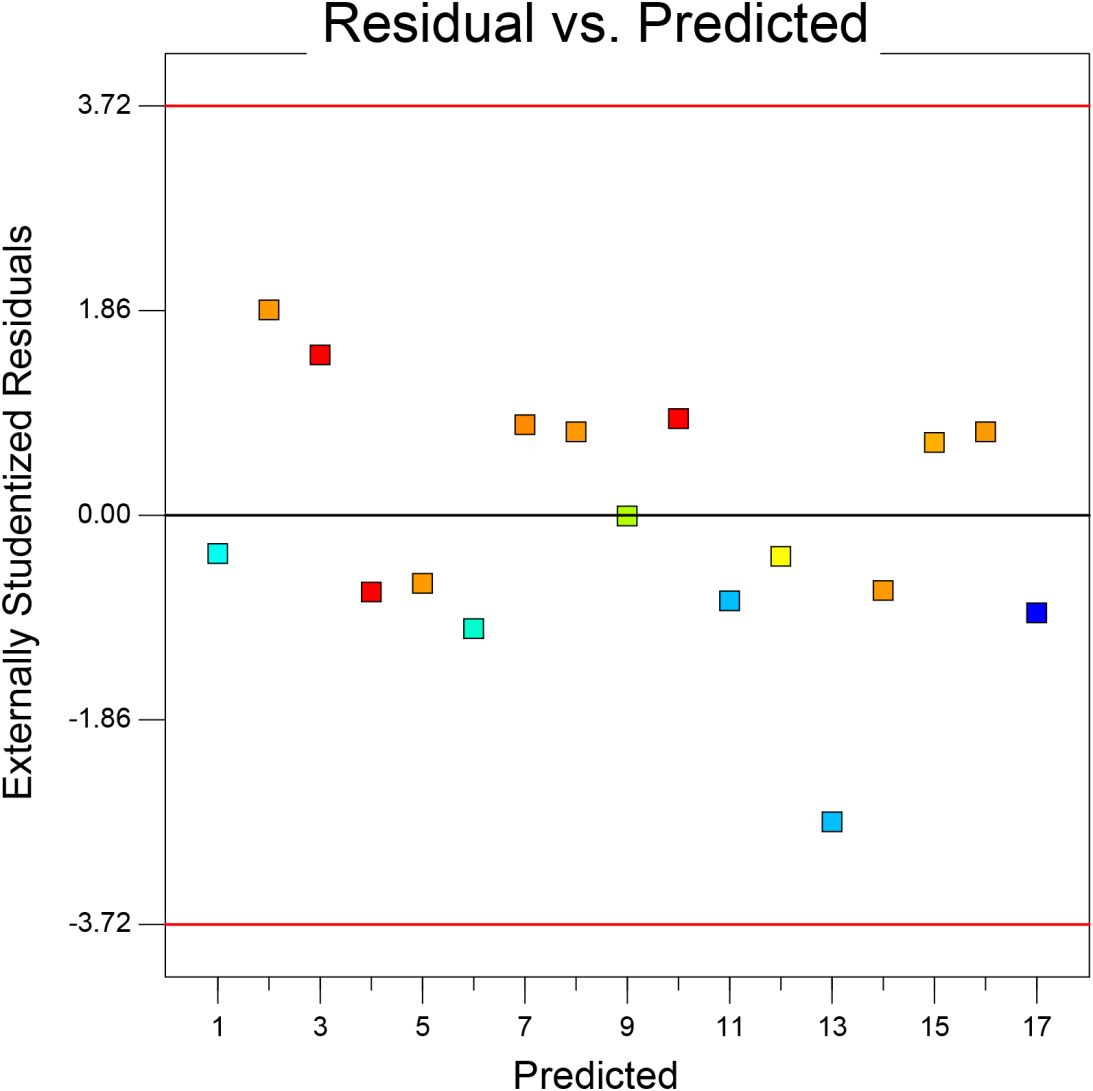
External studentized residual on emulsion stability

For a model to be used for predicting the experimental results, the predicted values versus actual plot must be randomly distributed along the slanting line of the graph and close to each other (Martin et al., 2017). Figure 1 displays a plot of the predicted values versus the observed values for emulsion stability. Approximately, data points were randomly distributed along the slanting line of the graph. In this study, since the consumption amounts of modified starch, corn oil, and gum rosin were different in the preparation of the formulation runs, the stability of the emulsions was different and the dispersion of points along the slanting lines was not very close to each other. Figure 2 reveals the plot of normal probability versus externally studentized residuals for the stability of emulsions. This plot was used to check the normality of the assumptions made. The above figure revealed that the residuals are normally distributed along the straight line, which confirms a linear relationship between the normal probability and the externally studentized residual. The plot of the residuals versus experiment run number for emulsion stability is presented in Figure 3. The assumption made from this test is that a good model is obtained when the data points are randomly spread within the confidence limit and no trends or patterns are observed. It can be seen from the plots, the design points of the residuals have been randomly distributed (constant range of residuals across the graphs) within the confidence limit of (−3.72, 3.72) (Figure 4). No data points were found out of the interval (−3.72, 3.72), suggesting that there were no outliers of the residuals for the model. Furthermore, no specific pattern of the residuals was observed in the graphs, suggesting a constant variability of the original observation for all values of the responses (Fouladitajar et al., 2015). These plots confirm the adequacy and reliability of the developed response model for predicting the stability process of emulsions.

### 4.2 Flow and dynamic properties

**FIGURE 5:**
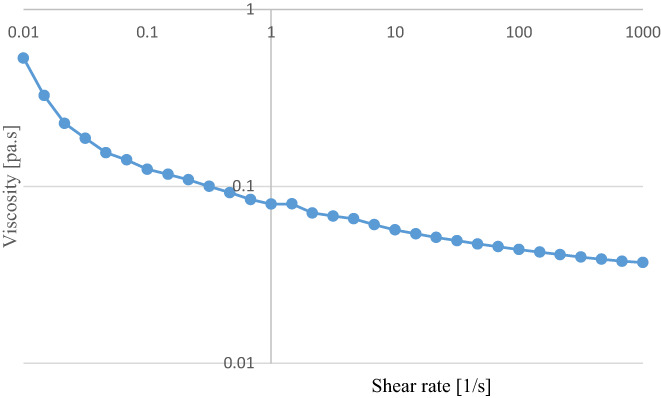
Viscosity changes of beverage emulsions at 2°C in the optimal sample

Determination of rheological properties is a common tool for specifying the emulsion stabilization mechanism, so that the information obtained can be used to change the system conditions for improving the performance or predicting stability. Different models have been used to evaluate the emulsion flow characteristics. Herschel–Bulkley model was the most suitable model to describe the flow behavior of the samples with respect to *R* ^2^ (coefficient of determination) and adjusted *R* ^2^. Newtonian fluids have a flow behavior index (*n*) equal to 1.0, while *n* < 1.0 represents shear thinning behavior, and flow behavior index >1.0 indicates shear thickening behavior (Anton et al., 2001). The consistency coefficient of the optimal emulsion sample was 0.46 Pa.s^n^ and the flow behavior index was 0.88. Since the flow behavior index was less than 1, it can be concluded that it is a non‐Newtonian fluid (Razavi et al., 2007). The apparent viscosity of the optimal emulsion sample was 0.0439 Pa.s. As depicted in Figure 5, the apparent viscosity has declined with the elevation of the shear rate, indicating that the emulsion has had a pseudoplastic behavior. In other words, the emulsion has a shear thinning behavior, which is described by the dominance of attraction forces over repulsion forces. Since the rheological features of the emulsions are affected by their components, so the use of gum rosin as a stabilizer can lead to a pseudoplastic behavior in emulsion (Taherian et al., 2006). The pseudoplastic characteristics of emulsions are probably due to their flocculation and deflocculation of oil drops. As the shear force increases, the deflocculation intensifies, and as a result, the viscosity of emulsions decreases. Small hydrodynamic forces are not able to break emulsions at low shear rates. As the shear rate grows, the hydrodynamic forces prevail, break the emulsions, and reduce the viscosity (Mandala et al., 2004). Generally, the research data showed that beverage emulsion is a pseudoplastic and time‐independent non‐Newtonian fluid.

**FIGURE 6:**
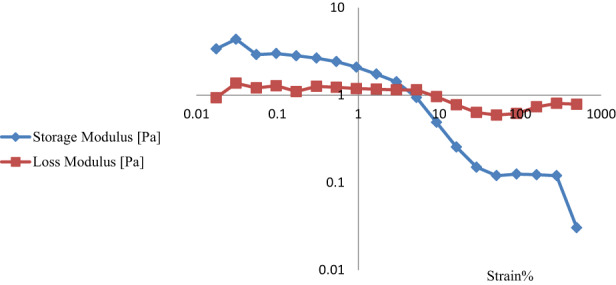
Strain sweep of modules G′ and G″ for the optimal emulsion

**FIGURE 7:**
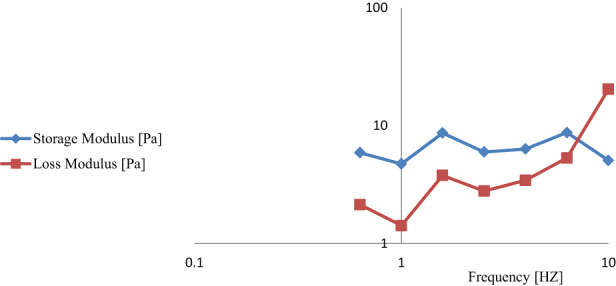
Frequency sweep of modules G′ and G″ for the optimal emulsion

Strain sweep test is the first step in analyzing the dynamic rheological properties during which the linear viscoelastic range is determined. The linear viscoelastic range is the region in which the values of the storage module and loss module are independent of strain. Meanwhile, in the nonlinear viscoelastic range, the values of these modules begin to drop with increasing strain. Figure 6 depicts the results of the strain sweep test for the optimum emulsion formula. The higher storage modulus than the loss modulus in the linear viscoelastic region indicates that the optimal emulsion behaves similarly to the viscoelastic solid. Consequently, corn oil had no clear effect on the viscoelastic behavior of beverage optimum emulsion, and the emulsifier (modified starch) played an important role in this property (Zhao et al., 2015). It was also clear that the strain range for the linear viscoelastic region was between 0.01% and 1%, after which storage and loss modulus decreased for the emulsion sample in the strain sweep test (nonlinear region). The value of τ_y_ is the sign of the first stress that causes the destruction of the structure whereby irreversible elastic property is observed. τ_y_ is the stress corresponding to the end of the viscoelastic linear region and is equivalent to the yield stress. Yield stress is a measure of the resistance of elastic and inelastic bonds between the structural units of a building to mechanical forces. In general, the higher the value of τ_y_, the greater the maintenance of viscoelastic structure and behavior within the applied strain range (Fomuso et al., 2001; Mezger, 2020). Frequency sweep test was performed within linear viscoelastic range to evaluate the viscoelastic behavior of emulsions. Typically, the storage modulus and loss modulus at the middle frequencies intersect each other and change the behavior of the system so that elastic behavior prevails at higher frequencies (Moreira et al., 2012). The pattern obtained from the frequency sweep test indicates the dependence of storage modules and loss modules on frequency (Figures 5 and 7). Approximately, the G′ and G″ modules are paralleled up to the frequency of 6.3 Hz, and the emulsion shows a linear viscoelastic behavior in the low and medium strain ranges, with the G′ modulus being higher than the G″ modulus within the entire strain range. However, the G′ modulus decreased slightly with increasing frequency. At low frequencies, the emulsion has the opportunity to reform the broken bonds in a frequency cycle. Hence, broken bonds are reformed which, while maintaining the structure, cause the emulsion to show its viscoelastic solid property. However, at high frequencies, the emulsion does not have the opportunity to reform its broken bonds, so the viscosity increased and the emulsion indicated the viscoelastic liquid property (Everett & McLeod, 2005). According to the polymer dynamics theory, for liquid‐like fluids, the frequency dependency of G′ values shows a power law connection (Ferry, 1980). Thus, the power law parameters used to model the frequency dependency of G′ was calculated using the following equation:

$$G^{\prime }={\mathrm{ɑ\omega}}^{b}.$$

where G′ is the storage modulus, ω denotes the oscillation frequency, and ɑ is a constant. The exponent b indicates the slope in a log–log plot of G′ versus ω. In the present study, the index ɑ was 4.74 and the index b for the optimal emulsion sample was 0.363. Index b shows the dependence of G′ on frequency. The tendency for a gel‐like structure will be greater for a smaller index b. In other words, low index b corresponds to elastic gels and an index b close to 1 indicates viscous gels. At b values near to zero, G′ does not change with frequency (Hesarinejad et al., 2014).

### 4.3 Effect of concentrations of modified starch, corn oil, and gum rosin on the creaming stability and opacity of the beverage emulsions

**FIGURE 8:**
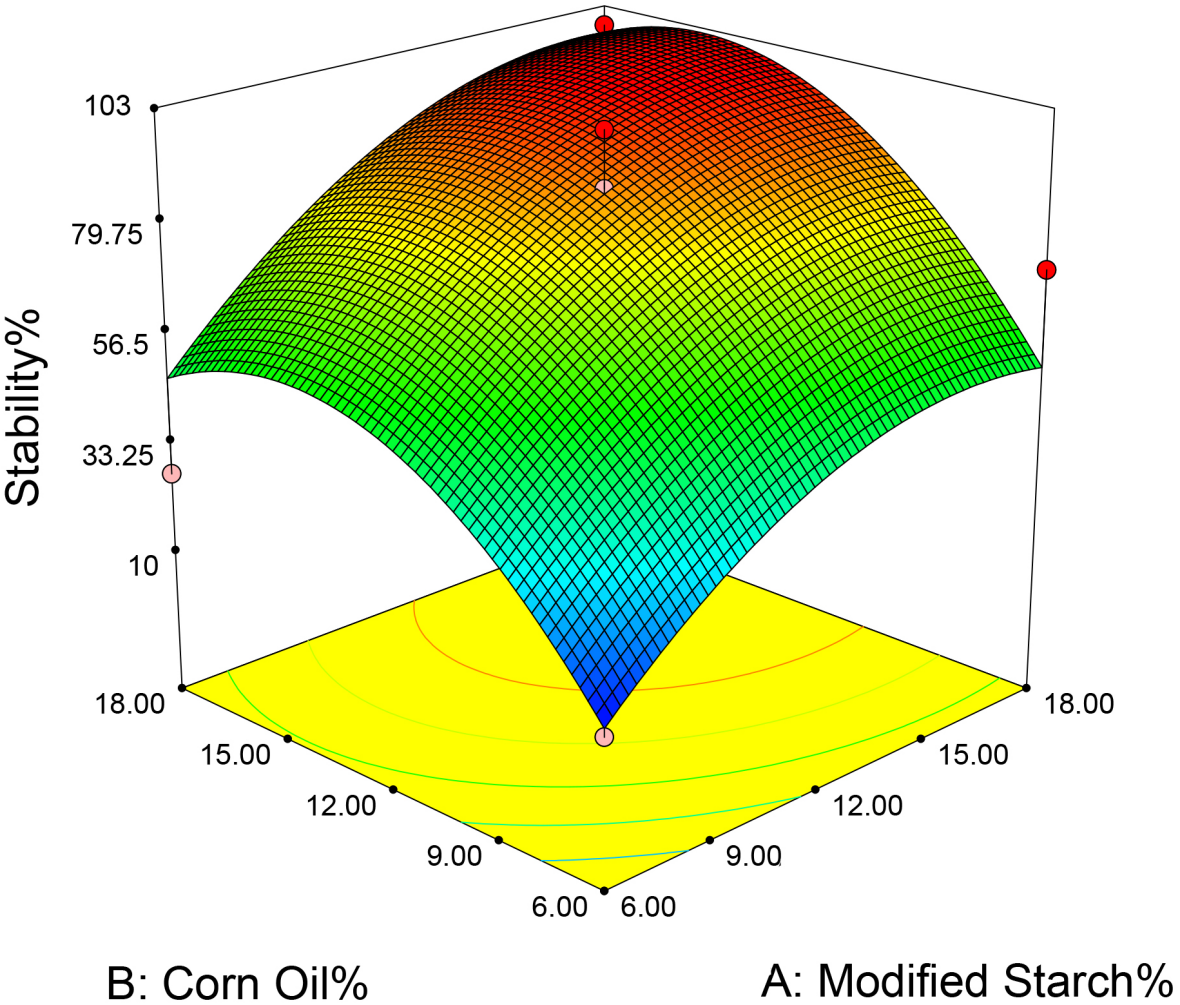
Response surfaces showing the effect of factors on the stability of optimal emulsion

**FIGURE 9:**
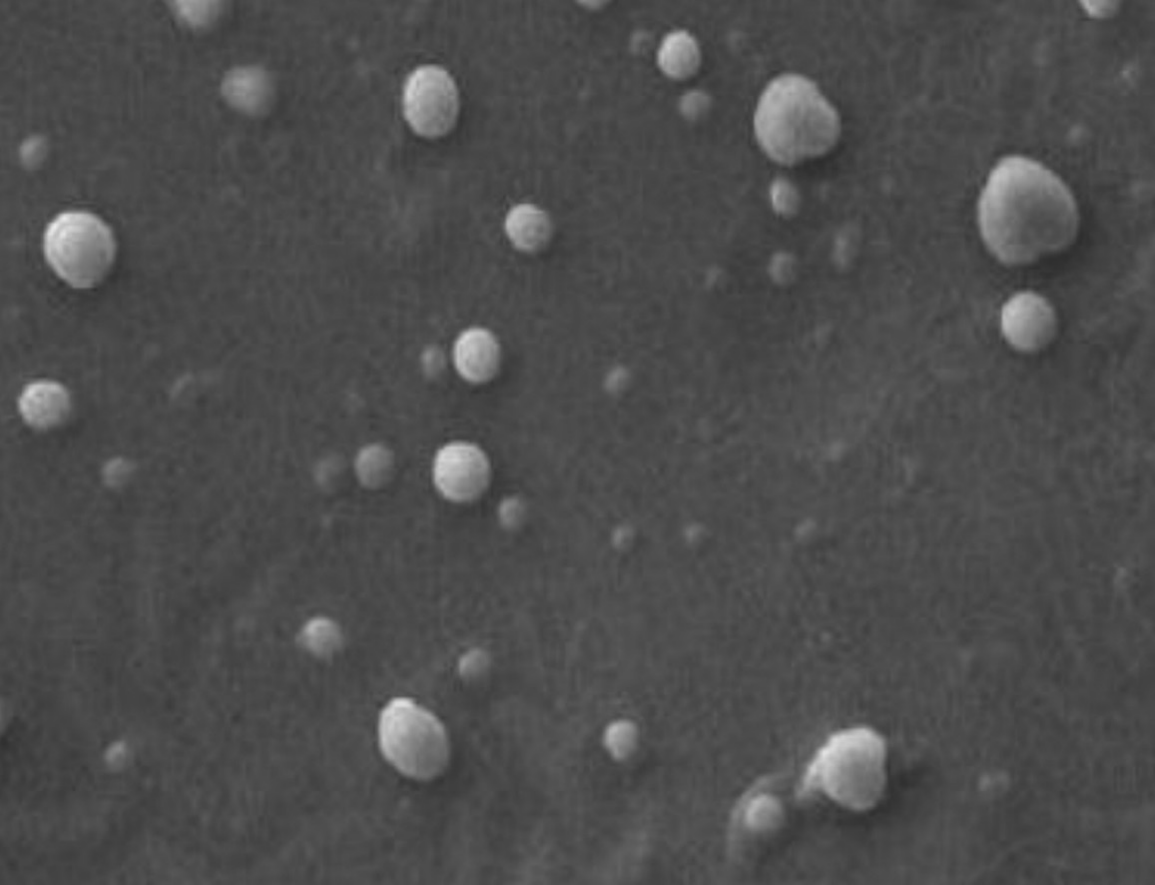
SEM image for optimal emulsion

Emulsion stability is very important in beverage preparation since many of these products require durability for weeks or months (Raikos et al., 2017). The first aim of this study was to identify the optimal concentration of modified starch for the formation of a protective film around the droplets and for long‐term stability of the emulsion (Figure 8). Visual observations and scanning electron microscope (SEM) images (Figure 9) showed that the starch used in the emulsion formulation at concentrations above 12% was sufficient to form a stable emulsion. Low concentration (6%) emulsions formed immediately once the homogenization process was exposed to gravitational separation by forming an obvious ring. Thus, it can be concluded that for modified starch at concentrations below 12%, the amount of starch was not enough to cover the surface of all droplets, and as a result, the instability of emulsions at low concentrations increased. Also, over time the increase in the diameter of the particles would accelerate their accumulation in the higher layers. Studies have shown that the average diameter of these particles is less than 15 μm and their microstructure is uniform. There is a direct relationship between viscosity and stability of an emulsion; elevation of the amount of hydrocolloids in the formulation leads to increased viscosity of the aqueous phase, thus reducing the movement of oil droplets in the emulsion environment, whereby the emulsion stability increases (Juntarasakul & Maneeintr, 2018).

**FIGURE 10:**
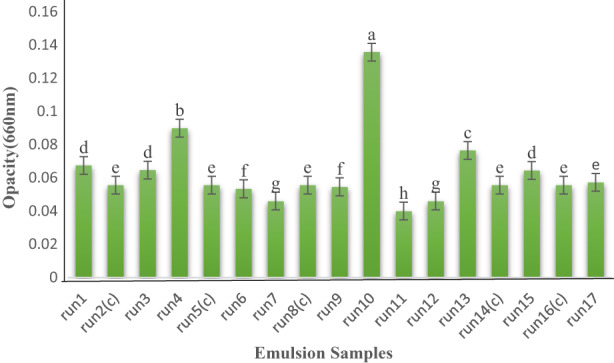
Opacity of emulsion samples prepared

Due to the increase in the amount of modified starch in the formulation, the creaming rate diminished significantly, which may be associated with an increase in the apparent viscosity of the beverage emulsion (*p* < 0.05). According to previous studies, enlargement of the molecular size of the dispersed phase (oil) and continuous phase (starch or hydrocolloid) of the formulation increases the viscosity, thus leading to emulsion stability. The droplets in the spherical phase are very close to each other in the continuous phase, so the oil droplets that make up the dispersed phase inevitably deform and appear spherically among the droplets of the continuous phase. They create strong bonds between the dispersed phase and the continuous phase, and finally establish the stability for the emulsified environment (Depree & Savage, 2001). The long‐term stability of the beverage emulsion depends on the ability of the absorbed emulsifier layer to prevent droplet accumulation during storage. This is related to the strength of repulsive forces and the surface charge of the droplets adsorbed in the monolayer (Raikos et al., 2017). Due to the creation of a barrier space among the droplets and entrapment of the droplets in the 3D lattice, the addition of hydrocolloids enhances the stability; these results were consistent with previous findings on the stabilizing characterization of modified starch (Najaf_Najafi & Fazeli, 2016; Yang et al., 2012). Corn oil concentration had no significant effect on increasing emulsion stability and creaming (*p* > 0.05). It seems that the difference in specific gravity of the dispersed phases and the continuous phase has reduced the emulsion stability. Gum rosin was used as a weighting agent in beverage emulsion formulations to boost stability by reducing the density difference between the oil and water phases. The high density of gum rosin (1.08 g/ml) increased the overall density of the oil phase and thus reduced the gravitational separation of the phases (Kanyuck, 2017). One‐way ANOVA showed that the independent variable gum rosin had no significant effect on creaming stability (*p* > 0.05). Previous studies have shown that adding gum rosin to oil phase could reduce stability emulsion, which was consistent with the findings elsewhere (Kaufman & Garti, 1984). Increased opacity indicates undesirable changes in the emulsion, but cloudy appearance is an important feature of citrus beverage, since the beverage obtained from such an emulsion is more like natural juice. Oil is the most important substance for creating a cloudy appearance plus opacity in beverages, and the particle size of oil droplets plays an important role in the stability of the beverage emulsion (Taherian et al., 2008). The opacity measured for emulsion samples is shown in Figure 10. In the study of multiple comparisons of the effect of oil concentration on opacity, there was a significant difference between concentrations of 5% and 9%, as well as 7% and 9% (*p* < 0.05). It confirms the positive effect of oil concentration on opacity. On the other hand, no significant difference was found between concentrations 5% and 7% (*p* > 0.05). The results of previous studies have also shown that elevation of the oil level in the beverage emulsion can significantly increase the opacity (*p* < 0.05) due to the scattering of a large portion of light (Taherian et al., 2008). Multiple comparisons of the modified starch concentration on the opacity indicated that there was a significant effect between concentrations of 6% and 18%, as well as concentrations of 12% and 18% (*p* < 0.05). Meanwhile, there was no significant difference between 6% and 12% concentrations (*p* > 0.05) (Table 6). Weighting agents are used as additives in the oil phase of some beverage emulsions to prevent of gravitational separation (McClements, 2004; McClements & Rao, 2011; Piorkowski & McClements, 2014). Previous studies have shown that by adding gum rosin to oil, opacity increases significantly (*p* < 0.05) (Kaufman & Garti, 1984); however, in this study, multiple comparisons of gum rosin concentrations revealed that gum rosin had no significant effect on opacity, which may be related to the difference in the type or concentration of materials used for producing the emulsion. These results were almost consistent with findings of other researchers.

### 4.4 Emulsion stability in model juice

Many food emulsions are exposed to heat treatment such as pasteurization, sterilization, and cooking during production, containing, and consumption. Thus, the strength and stability of the membrane around the droplets against the two‐phase, cohesion, and creaming is important (Gomez‐Díaz & Navaza, 2003). Emulsion stability in model juice can be different due to various factors such as differences in viscosity of the initial emulsion and diluted emulsion, the presence of particles in the concentrate, as well as chemical and physical interactions between emulsions and water compounds. Currently, oil ring quality measurement is one of the indices used in the beverage industry to evaluate the stability of beverage emulsions. Production emulsions used in fruit juice after pasteurization (70°C and 90°C) were stable in water for 1 month, and the heat treatment process did not result in the formation of a visible ring in starch emulsions stabilized in the model juice. Previous studies have also shown that beverage emulsions stabilized by starch are very stable even after pasteurization. The formation of thick films around oil droplets causes stability through ester repulsion. Also, the high stability of the starch stabilized emulsion in the model juice is due to the lower mobility of the interfacial layers (Chaudhari et al., 2015). The research findings concurred with the results of other researchers.

## 5 CONCLUSION

The optimum concentrations of modified starch, corn oil, and gum rosin (as independent variables) were determined as 12%, 5%, and 1%, respectively. Modified starch as an emulsifier had an effective role in creating a suitable texture and emulsion stability due to the absorption of water in the continuous phase. Elevation of the concentration of modified starch lowered the rate of creaming and the formation of oil ring, and enhanced the stability of emulsions. Also, the concentration of corn oil had a positive effect on the opacity of the emulsion and the resulting model juice. On the other hand, although gum rosin is used in beverage emulsion formulations as a weighting agent to reduce the density difference between the oil and water phases, in this research it did not have a significant effect on emulsion stability. Considering the scarcity and expensiveness of Arabic gum for preparing beverage emulsions and based on research findings, it seems that it is possible to use gum rosin as a weighting agent and modified starch as a desirable emulsifier in the preparation of these types of emulsions.

## AUTHOR CONTRIBUTIONS

J.S.‐Y. and S.N. contributed to the study conceptualization and methodology, validated the study, carried out data curation, investigation, and obtained the resources. F.M. formally analyzed the data. J.S.‐Y., S.N., M.H.E., F.M., and E.K.H. wrote the original draft, reviewed, and edited the manuscript. J.S.‐Y. contributed to the project administration, acquired funding, and visualized and supervised the study. All authors have read and agreed to the published version of the manuscript.

## CONFLICT OF INTEREST

The authors declare that they have no conflict of interest.

## Data Availability

Data available on request due to privacy/ethical restrictions

## References

[fsn33115-bib-0001] Anton, M. , Chapleau, N. , Beaumal, V. , Delépine, S. , & de Lamballerie‐Anton, M. (2001). Effect of high‐pressure treatment on rheology of oil‐in‐water emulsions prepared with hen egg yolk. Innovative Food Science & Emerging Technologies, 2(1), 9–21.

[fsn33115-bib-0002] Bohlin, L. (1980). A theory of flow as a cooperative phenomenon. Journal of Colloid and Interface Science, 74(2), 423–434. 10.1016/0021-9797(80)90211-8

[fsn33115-bib-0003] Buffo, R. A. , Reineccius, G. A. , & Oehlert, G. W. (2002). Influence of time–temperature treatments on the emulsifying properties of gum acacia in beverage emulsions. Journal of Food Engineering, 51(4), 341–345. 10.1016/S0260-8774(01)00076-0

[fsn33115-bib-0004] Chaudhari, A. , Pan, Y. , & Nitin, N. (2015). Beverage emulsions: Comparison among nanoparticle stabilized emulsion with starch and surfactant stabilized emulsions. Food Research International, 69, 156–163.

[fsn33115-bib-0005] Depree, J. , & Savage, G. (2001). Physical and flavour stability of mayonnaise. Trends in Food Science & Technology, 12(5–6), 157–163. 10.1016/S0924-2244(01)00079-6

[fsn33115-bib-0006] Everett, D. W. , & McLeod, R. E. (2005). Interactions of polysaccharide stabilisers with casein aggregates in stirred skim‐milk yoghurt. International Dairy Journal, 15(11), 1175–1183. 10.1016/j.idairyj.2004.12.004

[fsn33115-bib-0007] Ferry, J. D. (1980). Viscoelastic properties of polymers. John Wiley & Sons.

[fsn33115-bib-0008] Fomuso, L. B. , Corredig, M. , & Akoh, C. C. (2001). A comparative study of mayonnaise and Italian dressing prepared with lipase‐catalyzed transesterified olive oil and caprylic acid. Journal of the American Oil Chemists' Society, 78(7), 771–774.

[fsn33115-bib-0009] Fouladitajar, A. , Zokaee Ashtiani, F. , Dabir, B. , Rezaei, H. , & Valizadeh, B. (2015). Response surface methodology for the modeling and optimization of oil‐in‐water emulsion separation using gas sparging assisted microfiltration. Environmental Science and Pollution Research, 22(3), 2311–2327. 25182429 10.1007/s11356-014-3511-6

[fsn33115-bib-0010] Gomez‐Díaz, D. , & Navaza, J. M. (2003). Rheology of aqueous solutions of food additives: Effect of concentration, temperature and blending. Journal of Food Engineering, 56(4), 387–392.

[fsn33115-bib-0011] Hesarinejad, M. A. , Koocheki, A. , & Razavi, S. M. A. (2014). Dynamic rheological properties of *Lepidium perfoliatum* seed gum: Effect of concentration, temperature and heating/cooling rate. Food Hydrocolloids, 35, 583–589.

[fsn33115-bib-0012] Hosseini, A. , Jafari, S. M. , Mirzaei, H. , Asghari, A. , & Akhavan, S. (2015). Application of image processing to assess emulsion stability and emulsification properties of Arabic gum. Carbohydrate Polymers, 126, 1–8. 25933515 10.1016/j.carbpol.2015.03.020

[fsn33115-bib-0013] Juntarasakul, O. , & Maneeintr, K. (2018). Evaluation of stability and viscosity measurement of emulsion from oil from production in northern oilfield in Thailand. Paper presented at the IOP Conference Series: Earth and Environmental Science.

[fsn33115-bib-0014] Kanyuck, K. (2017). Weighting agent and flavor compound interactions: The impacts of concentration, chemistry, and oil composition for application in beverage emulsions.

[fsn33115-bib-0015] Kaufman, V. R. , & Garti, N. (1984). Effect of cloudy agents on the stability and opacity of cloudy emulsions for soft drinks. International Journal of Food Science & Technology, 19(2), 255–261. 10.1111/j.1365-2621.1984.tb00348.xCitations

[fsn33115-bib-0016] Krstonosic, V. , Dokić, L. , Dokic, P. , & Dapcevic, T. (2009). Effects of xanthan gum on physicochemical properties and stability of corn oil‐in‐water emulsions stabilized by polyoxyethylene (20) sorbitan monooleate. Food Hydrocolloids, 23(8), 2212–2218.

[fsn33115-bib-0017] Mandala, I. , Savvas, T. , & Kostaropoulos, A. (2004). Xanthan and locust bean gum influence on the rheology and structure of a white model‐sauce. Journal of Food Engineering, 64(3), 335–342.

[fsn33115-bib-0018] Maravic, N. , Seres, Z. , Nikolic, I. , Dokic, P. , Kertész, S. , & Dokić, L. (2019). Emulsion stabilizing capacity of sugar beet fibers compared to sugar beet pectin and octenyl succinate modified maltodextrin in the production of O/W emulsions: Individual and combined impact. LWT, 108, 392–399.

[fsn33115-bib-0019] Martin, J. , De Adana, D. D. R. , & Asuero, A. G. (2017). Fitting models to data: Residual analysis, a primer. In J. P. Hessling (Ed.), Uncertainty quantification and model calibration (Vol. 133). InTech. 10.5772/68049https://dx.doi.org/

[fsn33115-bib-0020] McClements, D. J. (2004). Food emulsions: Principles, practices, and techniques. CRC Press.

[fsn33115-bib-0021] McClements, D. J. , & Rao, J. (2011). Food‐grade nanoemulsions: Formulation, fabrication, properties, performance, biological fate, and potential toxicity. Critical Reviews in Food Science and Nutrition, 51(4), 285–330. 10.1080/10408398.2011.559558 21432697

[fsn33115-bib-0022] Mezger, T. (2020). The rheology handbook: For users of rotational and oscillatory rheometers: European Coatings.

[fsn33115-bib-0023] Moreira, R. , Chenlo, F. , Silva, C. , Torres, M. , Díaz‐Varela, D. , Hilliou, L. , & Argence, H. (2012). Surface tension and refractive index of guar and tragacanth gums aqueous dispersions at different polymer concentrations, polymer ratios and temperatures. Food Hydrocolloids, 28(2), 284–290. 10.1016/j.foodhyd.2012.01.007

[fsn33115-bib-0024] Najaf_Najafi, M. , & Fazeli, A. (2016). Evaluation of *Lepidium sativum* seed gum effect on physical stability and flow properties of oil‐in‐water emulsion prepared by high‐speed dispersing. Food Science and Technology, 14(64), 126–116.

[fsn33115-bib-0025] Peressini, D. , Sensidoni, A. , & de Cindio, B. (1998). Rheological characterization of traditional and light mayonnaises. Journal of Food Engineering, 35(4), 409–417. 10.1016/S0260-8774(98)00032-6

[fsn33115-bib-0026] Piorkowski, D. T. , & McClements, D. J. (2014). Beverage emulsions: Recent developments in formulation, production, and applications. Food Hydrocolloids, 42, 5–41. 10.1016/j.foodhyd.2013.07.009

[fsn33115-bib-0027] Raikos, V. , Duthie, G. , & Ranawana, V. (2017). Comparing the efficiency of different food‐grade emulsifiers to form and stabilise orange oil‐in‐water beverage emulsions: Influence of emulsifier concentration and storage time. International Journal of Food Science & Technology, 52(2), 348–358. 10.1111/ijfs.13286

[fsn33115-bib-0028] Ramaswamy, H. S. , Arora, J. K. , Vatankhah, H. , & Rattan, N. (2020). Effect of utilization of alternative hydrocolloid‐based stabilizers on rheology of oil‐in‐water beverage emulsions. Journal of Food Measurement and Characterization, 14(3), 1744–1753.

[fsn33115-bib-0029] Razavi, S. M. , Najafi, M. B. H. , & Alaee, Z. (2007). The time independent rheological properties of low fat sesame paste/date syrup blends as a function of fat substitutes and temperature. Food Hydrocolloids, 21(2), 198–202. 10.1016/j.foodhyd.2006.03.008

[fsn33115-bib-0030] Taherian, A. R. , Fustier, P. , Britten, M. , & Ramaswamy, H. S. (2008). Rheology and stability of beverage emulsions in the presence and absence of weighting agents: A review. Food Biophysics, 3(3), 279–286. 10.1021/jf0002903

[fsn33115-bib-0031] Taherian, A. R. , Fustier, P. , & Ramaswamy, H. S. (2006). Effect of added oil and modified starch on rheological properties, droplet size distribution, opacity and stability of beverage cloud emulsions. Journal of Food Engineering, 77(3), 687–696.

[fsn33115-bib-0032] Taherian, A. R. , Fustier, P. , & Ramaswamy, H. S. (2007). Effects of added weighting agent and xanthan gum on stability and rheological properties of beverage cloud emulsions formulated using modified starch. Journal of Food Process Engineering, 30(2), 204–224.

[fsn33115-bib-0033] Yang, J. S. , Jiang, B. , He, W. , & Xia, Y. M. (2012). Hydrophobically modified alginate for emulsion of oil in water. Carbohydrate Polymers, 87(2), 1503–1506. 10.1016/j.carbpol.2011.09.046

[fsn33115-bib-0034] Zhao, X. , Liu, F. , Ma, C. , Yuan, F. , & Gao, Y. (2015). Effect of carrier oils on the physicochemical properties of orange oil beverage emulsions. Food Research International, 74, 260–268. 10.1016/j.foodres.2015.05.002 28411991

